# Finding a new therapeutic approach for no-option Parkinsonisms: mesenchymal stromal cells for progressive supranuclear palsy

**DOI:** 10.1186/s12967-016-0880-2

**Published:** 2016-05-10

**Authors:** Margherita Canesi, Rosaria Giordano, Lorenza Lazzari, Maurizio Isalberti, Ioannis Ugo Isaias, Riccardo Benti, Paolo Rampini, Giorgio Marotta, Aurora Colombo, Emanuele Cereda, Mariangela Dipaola, Tiziana Montemurro, Mariele Viganò, Silvia Budelli, Elisa Montelatici, Cristiana Lavazza, Agostino Cortelezzi, Gianni Pezzoli

**Affiliations:** Parkinson Institute, G.Pini-CTO, exICP, Milan, Italy; Cell Factory, Unit of Cell Therapy and Cryobiology, Fondazione IRCCS Ca’ Granda Ospedale Maggiore Policlinico, Via F Sforza 35, 20122 Milan, Italy; Interventional Neuroradiology Unit, Fondazione IRCCS Ca’ Granda Ospedale Maggiore Policlinico, Milan, Italy; Julius-Maximilians-Universität Würzburg and Neurologische Klinik, Universitätsklinik Würzburg, Würzburg, Germany; Department of Pathophysiology and Transplantation, Human Physiology Section, Università degli Studi di Milano, Milan, Italy; Nuclear Medicine Unit, Fondazione IRCCS Ca’ Granda Ospedale Maggiore Policlinico, Milan, Italy; Neurosurgery Unit, Fondazione IRCCS Ca’ Granda Ospedale Maggiore Policlinico, Milan, Italy; Fondazione IRCCS Policlinico San Matteo, Pavia, Italy; Bone Marrow Transplantation Center, Fondazione IRCCS Ca’ Granda Ospedale Maggiore Policlinico, Milan, Italy

**Keywords:** Progressive supranuclear palsy, Mesenchymal stem/stromal cells, Cell therapy, Regenerative medicine

## Abstract

**Background:**

The trophic, anti-apoptotic and regenerative effects of bone marrow mesenchymal stromal cells (MSC) may reduce neuronal cell loss in neurodegenerative disorders.

**Methods:**

We used MSC as a novel candidate therapeutic tool in a pilot phase-I study for patients affected by progressive supranuclear palsy (PSP), a rare, severe and no-option form of Parkinsonism. Five patients received the cells by infusion into the cerebral arteries. Effects were assessed using the best available motor function rating scales (UPDRS, Hoehn and Yahr, PSP rating scale), as well as neuropsychological assessments, gait analysis and brain imaging before and after cell administration.

**Results:**

One year after cell infusion, all treated patients were alive, except one, who died 9 months after the infusion for reasons not related to cell administration or to disease progression (accidental fall). In all treated patients motor function rating scales remained stable for at least six-months during the one-year follow-up.

**Conclusions:**

We have demonstrated for the first time that MSC administration is feasible in subjects with PSP. In these patients, in whom deterioration of motor function is invariably rapid, we recorded clinical stabilization for at least 6 months. These encouraging results pave the way to the next randomized, placebo-controlled phase-II study that will definitively provide information on the efficacy of this innovative approach.

*Trial registration* ClinicalTrials.gov NCT01824121

## Background

Progressive supranuclear palsy (PSP), Steele–Richardson–Olszewsky syndrome (SR) type, is a progressive neurodegenerative disorder belonging to the group of taupathies, with motor, cognitive and behavioral symptoms. Its prevalence is about 6.5 cases per 100,000 people and its incidence is about 5.3 new cases every 100,000 people [1–3]. Although distinctive signs of PSP may appear already within the first 2 years of disease after onset, its clinical heterogeneity makes early diagnosis a challenge [4–6] and no reliable biomarkers are available. Therefore postmortem neuropathology is the diagnostic gold standard of PSP [7, 8]. Motor symptoms include gait disturbances, early onset of postural instability, backward falls, axial rigidity and restriction of vertical eye movements [9]. Personality changes and cognitive impairment are other frequent invalidating symptoms [10]. Quality of life deteriorates rapidly and patients are confined to a wheelchair a few years after the onset of disease [11]. Disease duration is generally no longer than 9 years [4, 10, 12, 13]. Aspiration pneumonia and respiratory failure are frequent causes of death [14]. PSP has all the negative features of the most severe neurodegenerative disorders: its etiology is unknown, it is invariably progressive, it does not respond to any available therapy and it brings a huge human and economic burden to society. Despite healthy neural cell replacement is the ideal objective of any curative therapy for PSP, as in many other neurodegenerative disorders, up to now no approach can efficiently achieve this goal. Therefore, even treatment that could reduce neural cell loss and stabilize clinical symptoms would be a significant breakthrough in this field. The use of several new drugs, such as davunetide, a tau-directed therapeutic agent, and donezepil, failed to exert beneficial effects in PSP patients [15]. Only slight improvement was achieved with coenzyme Q10 [16]. On the other hand, both preclinical in vitro investigations as well as preliminary clinical studies have shown that bone marrow (BM) derived mesenchymal stromal cells (MSC) may offer a new strategy for several neurodegenerative disorders [17–19]. The biological hypothesis underlying this approach is that MSC can exert neuroprotective effects by reducing cell apoptosis and neural cell loss [17]. We specifically define in our work MSC as mesenchymal stromal cells since they completely fulfil the minimal requirements set by ISCT for mesenchymal stromal cells [18], while they only partially comply with the definition of stem cells [19]. With all this in mind and with no intention of actually replacing diseased neurons, we conceived a phase I study to test the safety of MSC intra-arterial infusion, as well as its effects in slowing down the rate of progression of the disease in PSP patients. We followed up the enrolled patients for 1 year, with the best available validated clinical rating scales for the assessment of Parkinsonism, neuroimaging procedures and an automated biomechanical evaluation. In the present report we describe the one-year follow-up results obtained in the first five PSP patients treated with autologous MSC.

## Methods

### Protocol approval, patient screening and cell manufacturing

The protocol was authorized by the local Ethics Committee of Fondazione IRCCS Cà Granda Ospedale Maggiore Policlinico (Italy), by the national competent authority for phase-I cell therapy at the National Health Institute (Istituto Superiore di Sanità) and approved by the Italian Medicines Agency (Agenzia Italiana del Farmaco, AIFA). The trial is registered at ClinicalTrials.gov (NCT01824121). All patients gave their written informed consent. A detailed description of the study design, inclusion and exclusion criteria, BM collection, MSC isolation and administration along with clinical (motor and neuropsychological) and neuroimaging assessments have been previously reported [20]. In order to efficiently select the patients to be treated in the clinical trial, a pre-screening procedure was implemented. The patients who met the inclusion/exclusion criteria were therefore enrolled in a pre-clinical, validation study and underwent bone marrow aspiration to test the ability of their BM to give rise to MSC with the due quantitative and qualitative characteristics. In particular, the following specifications were set up to classify MSC cultures as compliant and therefore proceed towards expanding them for clinical use: (a) ≥1-fold expansion between passage 0 and 1 and between passage 1 and 2; (b) normal karyotype at passage 0, 1 and 2.

This validation study was implemented because there is sporadic evidence that MSC from patients affected by neurological diseases might differ somehow from those generated from normal healthy donors [21]. In this way we ensured that the patients were treated only with cells that could be identified as standard MSC complying with the universally recognized characteristics [18]. A maximum of 30 mL of BM was harvested. All the cell preparations passed the quality controls following good manufacturing practices (GMP) rules.

### Clinical and neuropsychological assessment

The patients underwent neurological examinations to assess motor function using the following scales: unified Parkinson’s disease rating scale [22] (UPDRS part-III, motor score), Hoehn and Yahr staging [23] (H&Y), PSP rating scale [12] (PSP-RS). These tests, together with mini mental state evaluation (MMSE, according to Folstein et al. [24]) were assessed at baseline and at each follow-up point (1, 3, 6 and 12 months after cell administration). The clinical conditions were classified as “stable” when the UPDRS and PSP-RS scores had not diminished by more than 30 % compared to baseline and the H&Y staging did not change at the defined time point.

### Neuroimaging

All patients underwent longitudinal neuroimaging assessments, using brain magnetic resonance imaging (MRI) (baseline, 24 h after cell administration and after 1 year), striatal dopamine transporter single photon emission computed tomography (SPECT) and positron emission tomography (PET) (both at baseline and after 12 months). Tropanic tracers labeled with Iodine-123 (FP-CIT) and 18F-Fluoro-2-deoxyglucose (Beta-CIT) were used for SPECT imaging and for PET/TC imaging, respectively. For SPECT, intravenous administration of 110–140 MBq of [^123^I] FP-CIT (Datscan, GE-Health, Amersham, UK) was performed 30–40 min after thyroid blockade (10–15 mg of Lugol solution per os) in all patients. The analysis was performed as already described [25]. A volumetric template of grey matter anatomic distribution, generated from the Montreal Neurological Institute MRI single participant brain atlas by applying a macroscopic anatomical method (automated anatomic labelling), was reoriented and reformatted to obtain a 2.64-cm-thick reference section. A template of eight irregular regions of interest (ROIs) was manually drawn on this section to assess the anatomical extent of striatal and occipital structures having both specific and nonspecific uptake of [^123^I] FP-CIT, respectively. This ROI template was also positioned on the reference SPECT section and adjusted on both striatal and occipital cortex. Moreover, striatal ROIs were also segmented into their anterior (caudate nucleus) and posterior (putamen) portions. Specific striatal dopamine uptake transporter (DAT) binding of [^123^I] FP-CIT was calculated in the whole striatum, putamen and caudate nucleus using the formula:

$$\left[ {\left( {\text{mean counts in specific ROI}} \right) - \left( {\text{mean counts in occipital ROI}} \right)} \right]/\left( {\text{mean counts in occipital ROI}} \right).$$

We also calculated putamen/caudate ratios for each subject. All patients underwent [18] F-Fluoro-2-deoxyglucose positron emission tomography scanning (FDG-PET) at rest, after intravenous injection of 170 MBq. Each acquisition included a computed tomography (CT) transmission scan of the head (50mAs lasting 16 s) followed by a three-dimensional (3D) static emission of 15 min using a Biograph Truepoint 64 PET/CT scanner (Siemens). PET sections were reconstructed using an iterative algorithm (OS-EM), corrected for scatter and for attenuation, using density coefficients derived from the low dose CT scan of the head obtained with the same scanner, with the proprietary software. Images were reconstructed in the form of transaxial images of 128 × 128 pixels of 2 mm, using an iterative algorithm, ordered-subset expectation maximization (OSEM). The resolution of the PET system was 4–5 mm FWHM.

### Biomechanical evaluation

Biomechanical evaluation was assessed at baseline and at six and 12 months after cell administration. Equipment and settings were previously described [26]. For this study in particular, two specific sets of parameters, one for standing and one for gait initiation, were automatically extracted by means of ad hoc algorithms. For standing, we measured the center of pressure (CoP) mean velocity and spatial displacement [27–31]. To examine gait initiation we focused on anticipatory postural adjustment [26, 32] (i.e. imbalance and unloading phases) and measured the following parameters: (1) the duration of both phases, (2) the antero-posterior (AP) and medio-lateral (ML) shift and velocity of the CoP, (3) the CoP mean length and velocity. Finally, we also measured the (4) length and (5) velocity of the first step. Spatial parameters were normalized on the basis of body height (%BH).

### Cell administration

The median cell dose was 1.7 (1.2–2.0) × 10E6 MSC/kg of body weight. One single administration was performed for each patient. Before cell administration, the patient underwent neuroleptoanalgesia and was monitored by an anaesthetist. MSC were administered by intra-arterial route [33], with modifications according to local equipment and local standards. Briefly: with Seldinger technique, catheterization was carried out via the right common femoral artery (or the left one in the event of difficulty in achieving arterial access) using a 6F Ultimum EV (St Jude Medical, MN, USA) introducer and a 5F Hinck or Simmons (Terumo Europe NV, Leuven, Belgium) diagnostic catheter. An angiographic study of the cervical and intracranial arteries was performed, with the support of an 0.035 in., 150 cm long hydrophilic guide (Terumo Europe NV, Leuven, Belgium). Subsequently, with or without an exchange manoeuvre, using a 260 cm exchange wire Easykit 0.35″ or 0.38″ (ab medica s.p.a., Lainate (MI), Italy), a 90 cm 6F Envoy XB guiding catheter (Miami Lakes, FL, USA) was used, after intravenous administration of heparin sodium (3000–5000 IU according to body mass) to reduce the risk of thromboembolism. The guiding catheter, flushed with heparinised saline, was positioned at the origin of both internal carotid arteries and at the origin of the widest vertebral artery. Once the guiding catheter was in place, a Rebar 027 (130 or 145 cm) or Rebar 018 (153 cm) microcatheter (ev3/Covidien, Irvine, CA, USA), steered by a 205 cm Transend EX 0.014 (Boston Scientific, Natick, MA, USA), was moved forward and upward into the internal carotid arteries just above the origin of the ophthalmic arteries and into the basilar artery. The MSC were then injected into the various districts, through the microcatheter, using a pump at 70 mL/h. The catheter was flushed periodically with heparinised normal saline solution.

## Results

Five patients were included and treated in the study. Nine were pre-screened and seven tested positive in the validation study with a good rate of MSC expansion. As expected, in view of the severity of the disease, another two patients were not enrolled because one died before MSC administration and the other one rapidly worsened and was no longer eligible at the time of MSC treatment. Clinical and imaging data are listed in Tables 1 and 2, respectively. All the patients were alive at 12 months, except one, who died 9 months after cell administration for causes not related to the treatment: she fell accidentally before the six-month assessment and was not able to come for the follow-up visits. Also the last patient did not come for the 6 month assessment due to hospitalization for rehabilitation. Therefore the latest follow-up point was 12 months for three patients and 3 months for the remaining two. All the evaluable patients had stable UPDRS scores at the last follow-up. The PSP-RS also demonstrated disease stabilization at 6 and 12 months in all the evaluable patients, except one (PSP08). Another important positive effect was recorded by H&Y staging, which remained stable over time. The results of biomechanical evaluation are shown in Table 3.

**Table 1:** Patients’ description

	Case 1 (PSP01)	Case 2 (PSP02)	Case 3 (PSP06)	Case 4 (PSP08)	Case 5 (PSP09)
Demographic data and cell dose					
Gender	M	F	F	F	F
Age (years)	60	66	65	65	68
Disease duration (years)	8	7	4	6	5
Cell dose (×10^6^/kg)	1.4	1.7	2	1.8	1.2
MMSE					
Baseline	27.49	28.27	25.49	24.27	25.53
1-month	27.49	25.27	26.49	24.27	28.53
12-month	26.49	25.03	na	21.27	na
H&Y					
Baseline	4/5	4/5	4/5	4/5	4/5
1-month	4/5	4/5	4/5	4/5	4/5
3-month	4/5	4/5	4/5	4/5	4/5
6-month	4/5	4/5	na	4/5	na
12-month	4/5	4/5	na	4/5	na
UPDRS III					
Baseline	47	38	47	31	42
1-month	45 (−4 %)	37 (−3 %)	36 (−23 %)	31 (0 %)	48 (+14 %)
3-month	47 (0 %)	49 (+29 %)	48 (+2 %)	39 (+26 %)	48 (+14 %)
6-month	45 (−4 %)	51 (+34 %)	na	40 (+29 %)	na
12-month	47 (0 %)	47 (+24 %)	na	40 (+29 %)	na
PSP-RS					
Baseline	37	53	52	36	57
1-month	41 (+11 %)	40 (−25 %)	46 (−12 %)	39 (+8 %)	n.a.
3-month	44 (+19 %)	39 (−26 %)	43 (−17 %)	46 (+28 %)	51 (−11 %)
6-month	47 (+27 %)	63 (+19 %)	na	52 (+44 %)	na
12-month	47 (+27 %)	57 (+8 %)	na	53 (+47 %)	na

Demographical data, cell dose, baseline and follow-up neuropsycological assessments by mini-mental state evaluation (MMSE) and clinical scoring using three different scales

For UPDRS and PSP-RS the values are reports as absolute value and percentage of variation from baseline (in brackets)

H&Y Hoehn-Yahr stage, UPDRS III Unified Parkinson’s Disease Rating Scale part III, PSP-RS PSP rating score, na not available

**Table 2:** SPECT and PET data

	Baseline	12-month
Case 1 (PSP01)	R striatum = 0.16	R striatum = 0.19
	L striatum = 0.14	L striatum = 0.16
	R caudate nucleus = 0.27	R caudate nucleus = 0.10
	L caudate nucleus = 0.17	L caudate nucleus = 0.23
	R putamen = 0.08	R putamen = 0.27
	L putamen = 0.10	L putamen = 0.09
Case 2 (PSP02)	R striatum = 0.49	R striatum = 0.34
	L striatum = 0.35	L striatum = 0.21
	R caudate nucleus = 0.59	R caudate nucleus = 0.48
	L caudate nucleus = 0.39	L caudate nucleus = 0.33
	R putamen = 0.42	R putamen = 0.1
	L putamen = 0.35	L putamen = 0.01
Case 3 (PSP06)	R striatum = 0.42	na
	L striatum = 0.60	
	R caudate nucleus = 0.54	
	L caudate nucleus = 0.73	
	R putamen = 0.30	
	L putamen = 0.43	
Case 4 (PSP08)	R striatum = 1.00	R striatum = 0.61
	L striatum = 1.15	L striatum = 0.72
	R caudate nucleus = 1.30	R caudate nucleus = 0.67
	L caudate nucleus = 1.46	L caudate nucleus = 0.72
	R putamen = 0.79	R putamen = 0.55
	L putamen = 0.86	L putamen = 0.65
Case 5 (PSP09)	R striatum = 0.37	na
	L striatum = 0.46	
	R caudate nucleus = 0.37	
	L caudate nucleus = 0.55	
	R putamen = 0.36	
	L putamen = 0.39	

Specific striatal dopamine uptake transporter (DAT) binding of [^123^I] FP-CIT, calculated in the whole striatum, putamen and caudate nucleus using the formula: $\left[ {\left( {\text{mean counts in specific ROI}} \right) - \left( {\text{mean counts in occipital ROI}} \right)} \right]/\left( {\text{mean counts in occipital ROI}} \right)$

**Table 3:** Biomechanical evaluation

(A)	Normal values	Case 1 (PSP01)			Case 4 (PSP08)		
	(10°–90° percentile)	Basal	6-month	12-month	Basal	6-month	12-month
*Standing*							
Ellipse area (mm^2^) %BA	0.06–0.66	0.08	0.33	0.18	0.34	0.55	0.18
Ellipse eccentricity	0.62–0.97	0.97	0.92	0.8	0.98	0.99	0.80
Axis length AP avg. (mm) %FL	0.20–8.86	3.05	4.21	3.35	9.27^a^	3.73	3.35
Axis length ML avg. (mm) %FL	0.12–3.66	2.37	2.35	1.21	0.12	1.54	1.21
Sway path (CoP) velocity avg. (mm/s)	6.5–18.11	7.26	7.62	6.57	9.41	19.43	6.58
*Gait initiation*							
Imbalance phase							
Duration (s)	0.29–0.53	0.61^a^	0.84^a^	0.49	0.36	0.43	0.36
AP avg. (mm) %FL	9.58–17.26	0.86^a^	2.14^a^	1.04^a^	4.48^a^	1.73^a^	0.32^a^
AP vel. avg. (mm/s)	58.83–112.61	3.32^a^	13.68^a^	2.78^a^	22.33^a^	8.31^a^	1.01^a^
ML avg. (mm) %FL	4.6–19.21	5.13	6.61	3.55^a^	4.12^a^	3.18^a^	3.78^a^
ML vel. avg. (mm/s)	32.15–144.77	17.55^a^	43.53	23.1	24.78^a^	15.71^a^	27.22^a^
Sway path (CoP) velocity avg. (mm/s)	96.34–178.07	19.38^a^	46.99^a^	26.25^a^	36.17^a^	29.45^a^	28.74^a^
Sway path (CoP) length (mm)	36.73–63.60	14.29^a^	18.75^a^	11.69^a^	16.04^a^	11.12^a^	10.35^a^
Unloading phase							
Duration (s)	0.23–0.47	1.45^a^	0.83^a^	1.29^a^	1.09^a^	0.76^a^	1.36^a^
AP avg. (mm) %FL	3.9–14.44	7.94	8.73	8.79	11.48	12.28	8.21
AP vel. avg. (mm/s)	7.1–92.4	11.31	61.16	14.38	19.00^a^	37.42	10.71
ML avg. (mm) %FL	29.15–61.84	33.1	43.08	36.68	54.34	45.05	37.47
ML vel. avg. (mm/s)	265.14–481.64	78.31^a^	143.24^a^	85.81^a^	150.63^a^	151.28^a^	85.31^a^
Sway path (CoP) velocity avg. (mm/s)	269.18–510.98	108.73^a^	150.99^a^	95.58^a^	160.86^a^	163.10^a^	95.63^a^
Sway path (CoP) length (mm)	79.6–169.24	99.07	119.54	111.61	138.33	113.69	115.61
Step phase							
First step peak velocity (mm/s)	1475.4–1874.1	486.52^a^	783.05^a^	604.83^a^	714.70^a^	498.68^a^	566.64^a^
First step length (%BH)	26.27–33.63	21.04^a^	24.44^a^	17.08^a^	14.34^a^	8.21^a^	16.92^a^

(B)	Normal values	Case 3 (PSP06)	Case 5 (PSP09)
	(10°–90° percentile)	Basal	Basal
*Standing*			
Ellipse area (mm^2^) %BA	0.06–0.66	0.49	329.68^a^
Ellipse eccentricity	0.62–0.97	0.98	0.91
Axis length AP avg. (mm) %FL	0.20–8.86	6.78	5.16
Axis length ML avg. (mm) %FL	0.12–3.66	4.31^a^	4.13^a^
Sway path (CoP) velocity avg. (mm/s)	6.5–18.11	133.81^a^	12.69
*Gait initiation*			
Imbalance phase			
Duration (s)	0.29–0.53	ne	0.77^a^
AP avg. (mm) %FL	9.58–17.26	ne	6.01
AP vel. avg. (mm/s)	58.83–112.61	ne	16.70^a^
ML avg. (mm) %FL	4.6–19.21	ne	5.15
ML vel. avg. (mm/s)	32.15–144.77	ne	20.02^a^
Sway path (CoP) velocity avg. (mm/s)	96.34–178.07	ne	31.29^a^
Sway path (CoP) length (mm)	36.73–63.60	ne	22.06^a^
Unloading phase			
Duration (s)	0.23–0.47	ne	1.00^a^
AP avg. (mm) %FL	3.9–14.44	ne	5.63
AP vel. avg. (mm/s)	7.1–92.4	ne	8.63
ML avg. (mm) %FL	29.15–61.84	ne	54.85
ML vel. avg. (mm/s)	265.14–481.64	ne	129.37^a^
Sway path (CoP) velocity avg. (mm/s)	269.18–510.98	ne	132.25^a^
Sway path (CoP) length (mm)	79.6–169.24	ne	129.71
Step Phase			
First step peak velocity (mm/s)	1475.4–1874.1	ne	596.99^a^
First step length (%BH)	26.27–33.63	ne	12.78^a^

(A) Patients with complete follow-up; (B) patients with only basal evaluation. Case 2 (PSP 02) was not evaluable (ne) because of dystonia

For abbreviations: see text

^a^ Patient’s parameters outside the range between 10 and 90° percentile of healthy control subjects’ values

### Patient clinical presentation

#### Case I (PSP01)

The first patient enrolled was a male who noticed motor impairment in his right arm at the age of 52. Diagnosis of PSP was made two years after the onset of symptoms, when gait difficulty, instability with falls, dysarthria, dysphagia and vertical gaze appeared and motor symptoms worsened. Brain MRI and ECD SPECT were also compatible with PSP. Five years later he needed a walker due to postural instability and backward falls. Dysarthria and dysphagia worsened over time. The patient was enrolled in this study after 8 years of disease. At the time of enrollment, neuropsychological evaluation was in the normal range with the exception of mild depression, irritability and anxiety. Brain MRI showed slight subcortical, fronto-parietal and mesencephalic atrophy. FDG PET showed bilateral hypometabolism in the frontal superior gyrus, anterior cingulate, caudatus and midbrain. Severe reduction in dopamine transporter binding in the striatum was evident at FP-CIT SPECT. Biomechanical evaluation revealed normal measurements for standing, but several alterations of gait initiation.

#### Case II (PSP02)

Female, at the age of 63 she began to complain of rigidity, bradykinesia and unstable gait. She complained above all of neck pain that did not respond to any symptomatic therapy. Two years after the appearance of the first motor symptoms, clinical symptoms, brain MRI and FP-CIT SPECT supported the diagnosis of PSP. One year later she was still able to walk without assistance, but falls were more frequent and mild dystonic posture in the left leg was evident. The patient took part in the study after 7 years of disease. Neuropsychological evaluation was in the normal range with the exception of a slight deficit in long-term verbal memory. FDG PET showed hypometabolism in the polar temporal area, and in the ponto-mesencephalic and midbrain areas. Severe reduction in dopamine transporter binding in the striatum was evident. The patient was unable to perform biomechanical analysis of movement because of apraxia involving her right leg.

#### Case III (PSP06)

The story of the third patient began at the age of 62 when she first noticed bradykinesia and mild depression. A few months later postural instability and falls appeared. Four years later she complained mainly of postural instability and difficulty in eye movements. The patient took part in the study after 4 years of disease. At the time of enrollment neuropsychological evaluations revealed mild depression and mild deficit in executive functions and visual-spatial abilities. Brain MRI showed mild encephalic and cerebellar cortical atrophy, and severe mesencephalic atrophy. Before MSC treatment, we were able to perform biomechanical measurement of standing only. In comparison to healthy controls, we found high values of ML displacement of CoP and high CoP velocity, thus suggesting great difficulties in maintaining the upright position.

#### Case IV (PSP08)

Female, at 61 years of age she complained of instability and occasional falls. Diagnosis of PSP was made 4 years later. The patient took part in the study after 6 years of disease. Neuropsychological evaluation showed deficit in cognitive and executive functions, visual-construction abilities and selective attention. Depression and anxiety were evident. Brain MRI showed fronto-parietal cortical atrophy, severe mesencephalic atrophy and mild cortical cerebellum atrophy. FDG PET showed severe hypometabolism in brainstem, moderate bilateral hypometabolism in the parietal lobe and slight bilateral hypermetabolism in fronto-orbital regions. Dopaminergic striatal innervation loss was remarkable bilaterally. Biomechanical evaluation performed before MSC infusion revealed only increased antero-posterior (AP) displacement of CoP, which was greater than normal values in the standing position. However, almost all parameters related to gait initiation were altered.

#### Case V (PSP09)

Female, at the age of 63 she began to complain of postural instability and retropulsion. Four years later, she presented with mild movement impairment, instability, akinesia at night, mild dysphagia, and irritability. She also reported hypothyroidism and had had left knee replacement. Diagnosis of PSP was made and the patient took part in the study after 5 years of disease. At the time of enrollment, brain MRI showed mild cortical, and severe subcortical and mesencephalic atrophy. Cerebellar cortical atrophy was also evident. FDG PET showed bilateral hypometabolism in frontal area, right insula and temporal area; mesencephalic and cerebellar hypometabolism was also evident. Neuropsychological evaluation showed normal cognition, but deficits in executive and attentional functions, and in visual-spatial ability. Biomechanical measurements during standing showed high values of CoP sway path, in particular in medio-lateral (ML) direction. Regarding gait initiation, the patient showed altered values of almost all parameters.

### BM aspiration, cell administration and short term (24-hour) follow-up

**Fig. 1:**
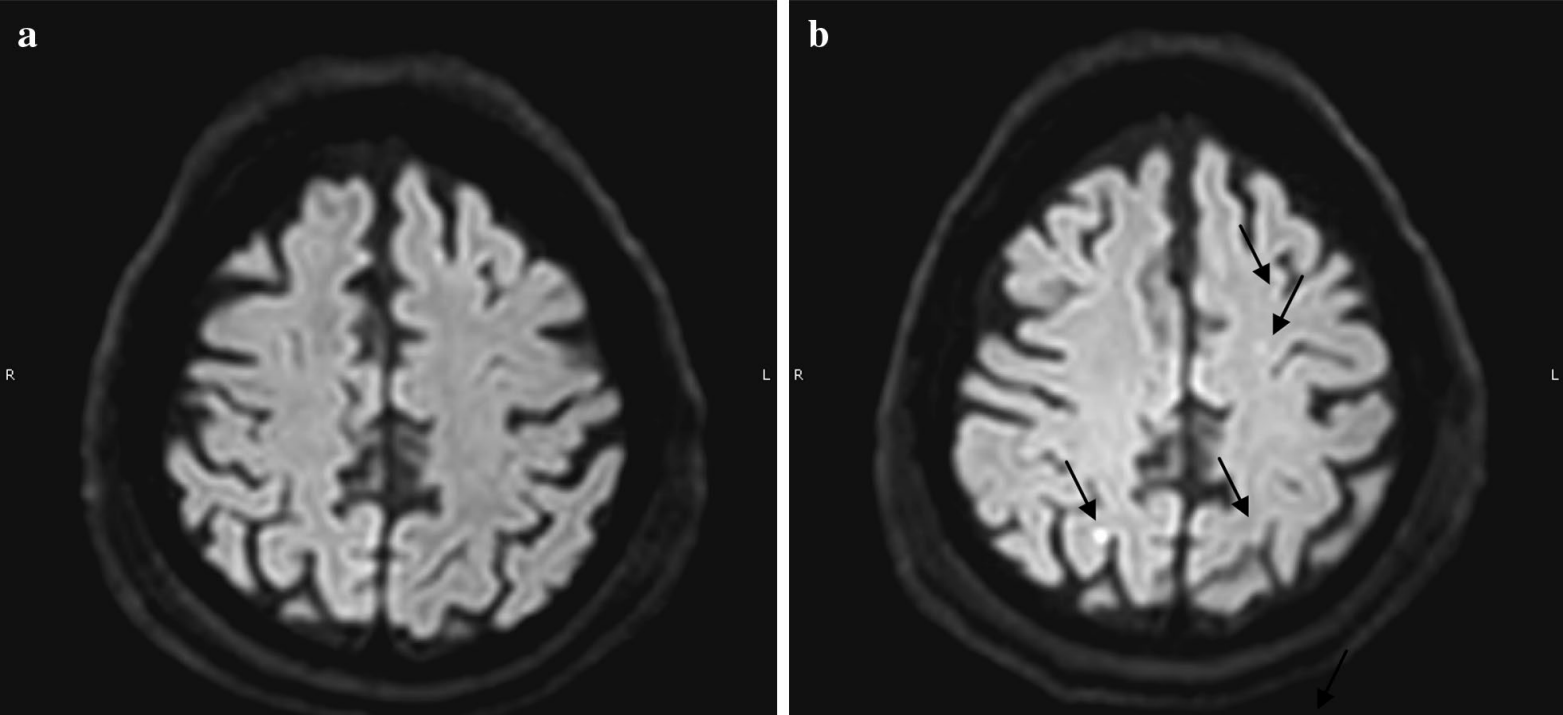
RMN study: representative figure of the RMN performed before **a** and 24 h after **b** cell administration. In **b** the *arrows* indicate several spotty lesions

All the patients underwent BM aspiration with no side effects. Cell administration was well tolerated in all patients. Neurological assessment remained stable after MSC administration in all patients, except one (Case V), in whom transient left hemiparesis was recorded. Also brain MRI performed 24 h after cell administration showed spotty ischemic lesions in all the patients (Fig. 1), while ischemic alterations in the posterior segment of the left inferior peduncle of the cerebellum and in the right mesencephalon were found in the last patient.

### Clinical assessment

#### Case I (PSP01)

One month after MSC treatment the patient and caregiver reported improvement in balance and gait, and a slight improvement in dysphagia. Neuropsychological evaluation showed no cognitive changes with regards to pre-treatment values and an improvement in mood.

At three, six and 12 month follow-up, clinical conditions were stable and the improvement in balance and gait persisted. Neuropsychological evaluation remained unchanged, with the exception (at 1 year) of mild daytime somnolence and worsening in executive and long-term memory (at the lower limit of the normal range). Mood was always in the normal range.

Biomechanical measurements performed 6 and 12 months after MSC infusion showed a global improvement in balance and gait initiation. In particular, the duration of the imbalance phase and the relative ML velocity of CoP normalized after MSC infusion.

#### Case II (PSP02)

At 1 month follow-up there were subjective improvements in stability, eye mobility, tone of voice and significant reduction in painful neck rigidity. The patient and her caregiver noticed an improvement in gait, although assistance was still necessary. Motor function remained stable for six months. Thereafter the patient and her caregivers noticed worsening of apraxia in the right leg resulting in instability and gait difficulty. Neck pain was still present, but somewhat milder than before MSC administration. Neuropsychological evaluation described worsening of executive function and long-term verbal memory.

Brain MRI showed increased atrophy in the mesencephalon, but no modification in other areas.

FDG PET findings were almost unchanged, with mild worsening in the prefrontal cortical area. The striatal density of dopamine transporters also worsened.

#### Case III (PSP06)

At one-month follow-up the patient, and her caregivers, reported improvement in gait and stability. Although she was not self-sufficient, she needed less assistance during daily activities, had improvement in ocular mobility mostly downward and reduction in photophobia. She also reported improvement in constipation. No changes for dysarthria and dysphagia were recorded. The improvement persisted at the 3 month follow-up visit.

Shortly before the 6 month follow-up evaluation, the patient fell and fractured her right foot. No biomechanical evaluation of posture and gait was thereafter attempted. Following this accident her clinical conditions worsened, the patient experienced depression and she refused food and drink. Renal function worsened and 9 months after MSC treatment the patient died in the emergency care unit due to cardiac arrest.

#### Case IV (PSP08)

One month after MSC administration neuropsychological evaluation showed global cognitive functions in the normal range, an increase in anxiety and depression. Her principal complaint was visual difficulty that was already present at the beginning of the disease. Three months after, improvement in global cognitive functions and increase in MMSE (from 24/26 to 27/30) was recorded. Nevertheless, depression and anxiety remained unchanged. Visual disturbances were still bothersome for the patient. Six months after MSC therapy subjective and objective evaluations were unchanged, the main complaint reported by the patient being ocular disturbances with photophobia and lacrimation, as at the onset of the disease. One year after MSC therapy, the clinical conditions of the patient were stable. FDG PET was unchanged, whereas FP-CIT SPECT showed a greater reduction in dopamine transporter binding in the striatum. A biomechanical evaluation of posture and gait initiation was performed 6 and 12 months after MSC infusion and showed global worsening of maintenance of upright posture and walking planning.

#### Case V (PSP09)

In the last patient, sensory-motor facio-brachial-crural left hemisyndrome appeared 12 h after MSC administration. After 24 h hyposthenia of upper left arm, hemi-facial paresis with severe dysarthria and dysphagia persisted. Brain MRI, performed 24 h after the procedure, showed ischemic alterations in the posterior segment of the left inferior peduncle of the cerebellum and in the right mesencephalon. During the following weeks, the neurological syndrome gradually improved with persistence of minor deficits (i.e. dysarthria, dysphagia and mild hyposthenia of the left arm). At 3 month-follow-up no sensory-motor deficits in the left arm were recorded. The patient did not attend the next follow-up visits, but information gathered on the telephone confirmed postural instability, whereas dysarthria was stable.

## Discussion

The “Holy Grail” for cellular therapy of degenerative disorders is neural cell replacement. However, despite initial encouraging results with fetal dopaminergic neuron transplantation [34], the goal is still far away and, up to now, no feasible, safe and effective cell therapy is available. On the other hand, there are numerous severe and progressive neurodegenerative disorders, which do not respond to any available therapy, symptomatic therapy included, and invariably lead to disabilities with heavy individual and societal consequences. In consideration of these still unmet clinical needs, major efforts are to be made to find innovative approaches that can provide novel tools to contrast disease progression and, at the very least, reduce the consequences of progressive neural cell loss.

With this aim in mind, we designed a phase I study, to test the safety of MSC administration in patients affected by PSP and to collect preliminary data on its efficacy. Herein we describe the evidence that we have gathered through first-in-man experience in five patients treated in the open phase of our trial and the findings during a one-year follow-up. To our knowledge, this is the first clinical trial testing autologous bone marrow MSC in PSP. The subjects in our trial were patients with rapidly progressing, severe disease for which there are no therapeutic options. Therefore, even though this phase I trial was not designed to test the efficacy of MSC, the stabilization of the rating scale scores after the intervention and during one-year follow-up is of utmost importance. Actually, in this study all subjects were evaluated by means of the best available rating scales for the assessment of motor function in patients with Parkinsonism (i.e. PSP-RS, UPDRS motor score and H&Y staging), as well as by biomechanical evaluation of gait and posture at different time points. We report that all the patients at the last follow-up had stable clinical assessment scores related to at least two validated scales and one patient maintained this stabilization for 1 year. Regarding the biomechanical evaluation, as expected, it confirmed the presence of great difficulties in balance and planning of gait [35] in all patients. Despite being not applicable to severely impaired patients, such a biomechanical evaluation proved to be a reliable method to investigate motor and postural capabilities in PSP patients. We were also able to describe mild improvement in one subject (PSP01) 12 months after MSC infusion.

Regarding safety, it must not be left unsaid that intra-arterial administration of MSC is associated with important safety concerns because of the intrinsic risk of microembolization, which, in our experience was invariably present in all the treated patients. This risk had been already reported by Lee and co-workers, who treated patients affected by multiple system atrophy [36] and seems to depend, to some extent at least, on the intrinsic infusion technique. It was indeed present also in the placebo group and at a higher frequency compared to the treated group (35 vs 29 %). Other factors that may be involved in microembolization during MSC intrarterial administration are cell size and type [37] and infusion velocity [38, 39]. In consideration of this risk and of its determinants, a stringent and accurate follow up was implemented, including frequent clinical assessments and the execution of MRI before and 24 h after intervention. An interdisciplinary evaluation of each single patient was performed jointly by the neurologist, the interventional radiologist and the anesthesiologist. In our study all the patients were alive 1 year after cell administration, except one, who died of the consequences of an accidental fall. To correctly interpret the significance of these findings, we analyzed the historical cohort of 455 PSP patients followed by our Center over the last years. In a subgroup of subjects (n = 118) with the same characteristics as the patients enrolled in the study, only 24 % of them were followed up for at least 1 year, the main causes of unavailability being death or severe disease progression (personal data, not shown). This makes the survival rate in our trial extremely significant.

## Conclusions

Despite their preliminary nature, these first-in-man results with PSP patients are encouraging and can be easily transferred to several other neurodegenerative disorders. The approach followed in our study is, in fact, not to replace diseased neurons (“replacement” cell therapy) but to reduce the consequences and the rate of neural cell deterioration by using MSC as a medication. The intention is to exploit their well-known biological function in preserving cell homeostasis and maintaining a healthy microenvironment (“rescue” cell therapy). Due to the complexity and the specialization of the different types of neural cells, a specific replacement cell therapy should be developed for any single disease, while the “rescue” cell therapy may be suitable for many types of neurodegenerative disorders. For all these reasons the experience herein reported may be of general interest as a way to find suitable therapy for orphan neurologic disorders. Moreover, it paves the way to the next phase-II randomized, double-blind, placebo-controlled trial that may provide more valuable insights into the potential efficacy of MSC for neurodegenerative disorders.
